# Medical Oncology Professionals’ Perceptions of Telehealth Video Visits

**DOI:** 10.1001/jamanetworkopen.2020.33967

**Published:** 2021-01-14

**Authors:** Arianna Heyer, Rachel E. Granberg, Kristin L. Rising, Adam F. Binder, Alexzandra T. Gentsch, Nathan R. Handley

**Affiliations:** 1 Sidney Kimmel Medical College, Philadelphia, Pennsylvania; 2 Department of Emergency Medicine, Thomas Jefferson University Hospital, Philadelphia, Pennsylvania; 3 Sidney Kimmel Cancer Center, Department of Medical Oncology, Thomas Jefferson University, Philadelphia, Pennsylvania

## Abstract

**Importance:**

Telehealth has emerged as a means of improving access and reducing cost for medical oncology care; however, use by specialists prior to the coronavirus disease 2019 (COVID-19) pandemic still remained low. Medical oncology professionals’ perceptions of telehealth for cancer care are largely unknown, but are critical to telehealth utilization and expansion efforts.

**Objective:**

To identify medical oncology health professionals’ perceptions of the barriers to and benefits of telehealth video visits.

**Design, Setting, and Participants:**

This qualitative study used interviews conducted from October 30, 2019, to March 5, 2020, of medical oncology health professionals at the Thomas Jefferson University Hospital, an urban academic health system in the US with a cancer center. All medical oncology physicians, physicians assistants, and nurse practitioners at the hospital were eligible to participate. A combination of volunteer and convenience sampling was used, resulting in the participation of 29 medical oncology health professionals, including 20 physicians and 9 advanced practice professionals, in semistructured interviews.

**Main Outcomes and Measures:**

Medical oncology health professionals’ perceptions of barriers to and benefits of telehealth video visits as experienced by patients receiving cancer treatment.

**Results:**

Of the 29 participants, 15 (52%) were women and 22 (76%) were White, with a mean (SD) age of 48.5 (12.0) years. Respondents’ perceptions were organized using the 4 domains of the National Quality Forum framework: clinical effectiveness, patient experience, access to care, and financial impact. Respondents disagreed on the clinical effectiveness and potential limitations of the virtual physical examination, as well as on the financial impact on patients. Respondents also largely recognized the convenience and improved access to care enabled by telehealth for patients. However, many reported concern regarding the health professional–patient relationship and their limited ability to comfort patients in a virtual setting.

**Conclusions and Relevance:**

Medical oncology health professionals shared conflicting opinions regarding the barriers to and benefits of telehealth in regard to clinical effectiveness, patient experience, access to care, and financial impact. Understanding oncologists’ perceptions of telehealth elucidates potential barriers that need to be further investigated or improved for telehealth expansion and continued utilization; further research is ongoing to assess current perceptions of health professionals and patients given the rapid expansion of telehealth during the COVID-19 pandemic.

## Key Points

**Question:**

How do medical oncologists perceive telehealth video visits for cancer care?

**Findings:**

In this qualitative study, in which 29 medical oncology health professionals were interviewed prior to the coronavirus disease 2019 (COVID-19) pandemic, the barriers to and benefits of telehealth video visits were elucidated. Although oncologists disagreed on the scope of a virtual physical examination and the financial impact of video visits, most recognized the benefit of eliminating travel and the challenge of delivering serious or bad news.

**Meaning:**

Differing opinions of medical oncology health professionals regarding the barriers to and benefits of telehealth video visits provide insight into the challenges that limit telehealth quality as well as its expansion potential.

## Teaser

This qualitative study surveyed physicians and other health professionals working in oncology to determine the perceived advantages and disadvantages of telehealth video visits in cancer care.

## Introduction

Telehealth is an emerging mode of care delivery that can improve access to care, reduce cost, and enhance patient and health professional experience while providing effective care.^1^ One central component of telehealth is the video visit, in which audiovisual technology is used to connect patients and health professionals in lieu of an in-person encounter.^2^ The video visit allows physicians to evaluate patients and provide treatment recommendations regardless of geographical distance. While physicians’ ability to perform a physical examination during the video visit is somewhat limited, many examination techniques are still feasible, and telehealth encounters and traditional in-person visits have similar performance characteristics.^3,4,5^

Telehealth has promise for patients with cancer, where concerns regarding access, cost, and experience are common.^6,7,8,9^ A randomized controlled trial of video vs in-person visits for follow-up after radical prostatectomy demonstrated equivalent efficiency, similar satisfaction, and significantly lower cost for the video visits.^10^ In addition, multiple studies have demonstrated that telehealth can improve access to cancer care for patients in rural settings while achieving equal or better patient satisfaction and generating cost savings when compared with in-person visits.^11,12,13^

Despite its many advantages, the use of telehealth in oncology is highly variable, and its uptake (until a recent surge due to the coronavirus disease 2019 [COVID-19] pandemic) has been limited nationally.^14^ Various factors likely contribute to slow adoption, including liability concerns, licensure challenges, reimbursement inconsistencies, and workflow ambiguity.^15^ We hypothesize that the perceptions health professionals have of this care delivery model are also a critical component of adoption, as the presence of perceived barriers to a service is associated with low utilization.^16^ However, to date, the perceptions of health professionals about the utility of telehealth for cancer care are not well understood.

The goal of this study is to report the results of a qualitative interview study focused on eliciting medical oncology health professional perceptions regarding the use of telehealth for patient care prior to the COVID-19 pandemic.

## Methods

### Study Setting

This study was conducted at Thomas Jefferson University, an urban academic health system in Philadelphia, Pennsylvania, with a cancer center that serves over 8000 patients each year. The practice is composed of 55 medical oncologists and 39 advanced practice professionals (APPs). In 2015, Jefferson launched JeffConnect, an enterprise-wide telehealth program that facilitates video visits for patients across a variety of use cases, including both scheduled visits and on-demand visits. Health professionals received training in the use of JeffConnect with both personalized guidance and online training modules. All Jefferson health professionals, including those in medical oncology, were incentivized (with relative value unit equivalents and an end-of-year bonus for high usage) to utilize video visits. This study was designed and conducted by a team consisting of an emergency medicine physician researcher with expertise in qualitative methods (K.R.), 2 medical oncology physician researchers (A.B. and N.H.), a research coordinator with expertise in qualitative methods (A.G.), and 2 MD/MPH candidate research assistants (A.H. and R.G.).

### Study Participants

All medical oncology health professionals (physicians and APPs) at Thomas Jefferson University were eligible to participate. The aim of our volunteer and convenience sample selection was to include health professionals with various levels of experience with telehealth video visits, as the objective of the study was to obtain medical oncologists’ perceptions regardless of their frequency of offering or engaging in video visits. Potential participants were recruited first in a faculty meeting led by N.H. and A.B., who then emailed all health professionals instructing those interested to respond directly to nonclinical team members (A.G., R.G., and A.H.) to maintain anonymity among medical oncology colleagues. Health professionals who did not respond to the initial email were recruited directly via email and phone by R.G. and A.H. The study was approved by the Thomas Jefferson University institutional review board, and verbal consent was obtained from each participant. Interview quotations were scrubbed to remove identifying information.

### Data Collection

We developed a semistructured interview guide designed to elicit health professionals’ perceptions regarding the utility of and barriers to delivery of telehealth services in cancer care (eAppendix in the Supplement). The initial interview guide was drafted by 1 member of the research team (N.H.) and based on previous literature on perceptions of telehealth. All other team members then contributed to and revised the draft until it reached its final form. Interviews were structured to be 1-on-1 to limit bias and influence that could result from larger interview groups with multiple participants. We targeted completion of 30 overall interviews, with a goal of 20 physician interviews and 10 APP interviews, with interviews continued until thematic saturation was reached. The interviews were conducted from October 30, 2019, to March 5, 2020, by 2 trained interviewers (R.G. and A.H.) via telephone or in person and were approximately 20 minutes in length. Health professionals were also asked to complete a demographic survey following the interview with questions regarding age, gender, race, number of years in practice, previous experience with telehealth, and specialty within oncology.

### Data Analysis

Interviews were audio recorded and transcribed. Transcripts were verified for accuracy, identifying information was removed, and results were imported into NVivo 12 version 12 (QSR International) for coding and analysis. Given the exploratory nature of this study, the codebook was developed using a conventional content analysis approach, a common method of analysis when coding categories are derived directly from data.^17^ All members of the research team read the first 3 transcripts and identified concepts that captured health professionals’ perceptions. Team members discussed the identified concepts to create an initial code structure. This was applied to subsequent interviews by 2 coders (R.G. and A.H.) and refined to include new themes as they emerged. This process was iterative and continued until coders and at least 1 other team member (A.G.) agreed upon a final coding structure, with discrepancies resolved through consensus. The 2 coders applied the final structure to all transcripts. Coders double coded 43% of the transcripts. We calculated intercoder reliability in NVivo 12 by using the κ coefficient.^18^

We organized themes and quotes into the 4 National Quality Forum (NQF) domains of clinical effectiveness, patient experience, access to care, and financial cost or impact on patients.^1^ Each table corresponds to 1 domain, and reveals positive and negative themes with accompanying participant quotes. We used descriptive statistics to characterize the study population based on information collected from the demographic survey. This qualitative study is reported according to the Consolidated Criteria for Reporting Qualitative Research (COREQ) reporting guideline.

## Results

Analysis of intercoder reliability in this study showed a near perfect agreement (mean κ = 0.86). Supporting this result is the percentage of agreement analysis, which revealed a mean of 98.7% (spread, 86%-100%) agreement of all codes.

We enrolled a total of 29 health professionals, including 20 physicians and 9 APPs (Table 1). The mean (SD) participant age was 48.5 (12.0) years, over half (15 [52%]) were women, and most (22 [76%]) were White. An equal proportion (11 [38%]) reported 5 or less years in practice and 21 or more years in practice. Most participants specialized in either liquid malignant neoplasms (14 [48%]) or solid malignant neoplasms (11 [38%]). Over half of respondents (15 [52%]) reported having used telehealth for at least 10 visits, while 3 (10%) reported no prior use of telehealth.

**Table 1.** Characteristics of Study Participants

Characteristics	Participants, No. (%)
Age, mean (SD), y	48.5 (12.0)
Sex	
Men	14 (48)
Women	15 (52)
Ethnicity	
White	22 (76)
Black	2 (7)
Other^a^	5 (17)
Years in practice	
≤5	11 (38)
6-10	3 (10)
11-20	4 (14)
≥21	11 (38)
Experience with telehealth	
None	3 (10)
≤10 visits	11 (38)
>10 visits	15 (52)
Specialty in oncology	
Solid tumor	11 (38)
Liquid tumor	14 (48)
General oncology	4 (14)

^a^ Included Asian, American Indian, and Native Hawaiian/Pacific Islander.

### Perceptions of Clinical Effectiveness

Respondents had divergent thoughts on the clinical effectiveness of telehealth for oncologic care (Table 2). Specifically, health professionals had opposing opinions on the capabilities of a virtual physical examination. Some reported they could not examine a sore throat, graft vs host disease, or shortness of breath via telehealth; others stated that they could assess the mouth and skin, as well as respiratory distress. Health professionals who noted the limitations of physical examinations on telehealth cited the dependency on patient knowledge, and raised concerns that the discordance between the physical examination and patient history could cause potentially important missed findings. Respondents noted that the lack of effective physical examinations made telehealth inappropriate for a number of visit types, including first appointments, patients who are seen only every 6 months to 1 year, multiple successive encounters, and patients who are symptomatic or sick. Another practical limitation recognized by 1 respondent was the inability to provide written information explaining complex treatments, referrals, and labs slips.

**Table 2.** Health Professionals’ Perceptions of Clinical Effectiveness

Theme	Quote (Participant ID—Telehealth utilization status^a^)
Positive	
Physical examination capabilities^b^	“You can certainly still assess somebody’s level of distress. You can assess their skin. There are things where talking to people that are really the telehealth pros where you can manage holding the camera and the right way to look in their eyes and their mouth.” (D07—higher utilizer)
More frequent patient interactions	“I think that you could have more frequent visits with your patient. … Instead of saying, ‘I know it’s hard for you to get here, come back in two weeks instead of one,’ it’s, ‘I’ll talk to you by video next week and I’ll see you the week after,’ and a lot of providers do that.” (A05—higher utilizer)
	“It would allow you to likely have frequent touch. … So I think that the frequency—high-risk patients, for example, so-called—try to anticipate the potential admissions, visits to the emergency room and so on.” (D01—lower utilizer)
Availability of laboratory tests	“So the other big convenience I think for the patient is when you have a video visit, the patient necessarily has to get their labs the day before so you’ll have them for the visit. So that … they’re typically all resulted by the time you speak with them. So the patient can hear about their labs and all of the results at the time of the visit.” (A05—higher utilizer)
Communicable disease	“The other thing that I think would be a benefit would be people that have acute illness that we don’t want in our office. Like if somebody thinks they have shingles, I would much rather have them as a video visit, because it’s a danger to the other patients in the office.” (D04—lower utilizer)
Negative	
Physical examination limitations^b^	“I think if you need to see … listen to someone’s lungs because they’re complaining of a cough or they have a sore mouth or something, you can probably get a pretty good picture, but it’s hard to look at a TV monitor and see a mouth, and you can’t listen to lungs and do that.” (A01—lower utilizer)
Physical examination and history discordant	“You can be doing a physical exam and they tell you, ‘Oh, no, I’m not in any pain or discomfort.’ You go to palpate the abdomen and then they flinch. It’s like, oh, well, talk to me about that. ‘Oh, yeah, you know what, two days ago I woke up with this pain on my side,’ and again, the door opens. There it goes—room for conversation. So in doing a video visit, we would have probably just ended it like, okay, no pain or discomfort … because I obviously wouldn’t be able to examine the abdomen.” (A08—no telehealth experience)
Physical examination limited to patient's knowledge	“There are situations where we actually see something that the patient is not reporting, so unless it is something that is visual in terms of what we observe on the video … if someone has a lump on their back and they don’t know about it, you’re not going to see that on a telehealth visit.” (D01—lower utilizer)
Restrict successive video visits secondary to limited physical examination	“I’d be very hesitant to let many successive video visits go by without actually laying hands on a patient. Right? Because we’re not doing physical exams, and so I don’t think—at least in what I do, which in no small part is making sure that patients don’t have recurrence—that we could get away … I just can’t see how it would be adequate medical care to do multiple successive visits without actually laying hands on a patient.” (D19—lower utilizer)
Inability to provide written information	“I can’t write things down for them and I do that extensively at my visits because I’m often giving them complex treatment plans, estimating survival, giving them options for different things they can do. And I’m making them appointments for a lot of different things, to see other doctors, to get tests done. Giving them lab slips, I can’t do that. You can’t hand somebody their lab slip over a video visit.” (D04—lower utilizer)

^a^ A higher utilizer was defined as a health professional who, at the time of the interview, had conducted more than 10 video visits. A lower utilizer was defined as a health professional who, at the time of the interview, had conducted 1-10 video visits. The participant ID has a 2-digit number, and either an “A” denoting an advanced practice professional, or a “D” denoting a physician.

^b^ A subtheme and associated quote that was identified as both a positive and negative feature of telehealth in oncology.

Other respondents found merits in the clinical effectiveness of telehealth. Some believed telehealth would allow for increased frequency of patient interactions, noting that for particularly high-risk patients, telehealth could be utilized to anticipate potential emergency department visits or hospital admissions. One respondent noted the potential use of telehealth for patients with communicable diseases, such as shingles, which may pose a risk to other patients in the office setting. Another respondent noted the utility of telehealth because laboratory tests were typically performed the day prior to the video visit, which allowed for results to be discussed at the time of the visit. This is in contrast to in-person encounters, where tests were often obtained by a phlebotomist immediately prior to examination.

### Perceptions of Patient Experience

Many respondents were concerned with the patient experience of telehealth, often noting the relationship between health professionals and patients as uniquely important in oncology (Table 3). Respondents frequently shared concern that a decreased ability to bond with and support a patient through telehealth is a disservice. Another concern was that patients may experience difficulty comprehending complex treatment discussions that may be better facilitated through an in-person interaction. Several respondents noted that it was not feasible to have discussions regarding serious or bad news through telehealth. A few respondents attributed this to potential technology glitches that made these conversations inappropriate. Respondents also reported that patients had expectations of a physical examination that may not be met when conducted via telehealth, potentially leaving the patient feeling unsatisfied.

**Table 3.** Health Professionals’ Perceptions of Patient Experience

Theme	Quote (Participant ID—Telehealth utilization status^a^)
Positive	
Patient follow-up	“A follow-up visit, the patient in the office today may come up with two or three questions, but then as they drive home all of a sudden they realize they have actually ten more questions and now they have to wait. So I think that that gives you … again, it’s more frequent touching, more frequent conversations, more frequent discussions. I think that patients … I’m sure they will love that part.” (D01—lower utilizer)
Eliminates complications associated with transportation	“They would prefer to have a video visit and not have to travel. Whether it be distance, or it may have been difficult for them to get out, maybe they’re more homebound and transportation issues may be in place for them. Or having that social support available for someone to bring them, whatever. It just … it works good for them and it works for us.” (A04—lower utilizer)
Multiple family members included	“Maybe accessibility to other family members. Sometimes they can’t always make it in with their loved ones due to work schedules or if they’re at home watching their children or whatnot, so if we had this planned out ahead of time on this day [or] at this time, maybe they could make it a point to be with the patient and then this way any other questions or concerns on their behalf can all be attended to and talked about at that one very moment instead of doing the call with the patient and then an hour later a phone call comes in from the daughter … because they misunderstood this and we get a chain of events.” (A08—no telehealth experience)
Family education and counseling	“Certainly educational things that could be done over video instead of, again, face-to-face or as an adjunct where the patients and their families—for example, stem cell transplant [recipients] have had the opportunity to educate themselves a little bit more and then come see me in person, because then it’ll be a much more valuable session. And it could be a telemedicine visit, because we could have a nurse or a nurse practitioner or a physician’s assistant triaging the visit or participating in the visit to answer questions too. So it might also turn out to be a more efficient way to use our physician extenders also.” (D14—higher utilizer)
Negative	
Comprehension difficulties	“It’s not helpful when someone really doesn’t understand their treatment or has questions and they just can’t get it through talking. They need to be physically in front of you. Because that sometimes that seems to help more in understanding things.” (A03—lower utilizer)
Patient-health professional lose connection	“Cancer care, particularly a new diagnosis of cancer, is not something that most patients are gonna wanna handle through a video visit. They want to get to know you. They want to see you. They want to see your body language. They want to be able to get a relationship with their doctor. And you generally speaking can’t forge a good relationship with a patient just on video visits.” (D13—higher utilizer)
	“I think you lose a little bit of the personal connection you get with people when you sit in the same room and you talk to them and you lay your hands on them and you do an exam. The person who was in [City in PA 1], even though we saw her in the video visits and we did all that, she said that she didn’t get the services she needed, she didn’t feel like we were connected to her, she felt like she was alone.” (A01—lower utilizer)
Inappropriate for sensitive conversations	“And it’s also very hard to break bad news when not in-person. So if it’s gonna be something like—if it’s gonna be a very serious conversation, it’s not feasible to do it via telehealth—I don’t think that’s really fair or warm. So that’s just an inherent flaw in telemedicine.” (D12—higher utilizer)
Technology problems make it inappropriate for sensitive conversations	“It’s still glitchy, it doesn’t always work. So, I think having hard conversations via telehealth is always not really appropriate. And a lot of my conversations, unfortunately, are hard, a lot of my patients are pretty sick and their disease and things can get complicated.” (D20—higher utilizer)
Patient desire for physical examination	“Patients feel like they need a good exam sometimes. Well, you can’t do that by video. That’s when I said patients will be like, ‘Well, I’ve done two videos. Let me come in and get examined.’” (A05—higher utilizer)
	“You can’t do a physical exam, that’s a huge one. And that’s such an important part of a doctor visit. The patients kind of feel cheated, they don’t feel like it’s a real visit if I don’t put my hands on them. Sometimes I will do an exam that I don’t really have to, I’ll listen to their heart and lungs because that’s the ritual, patients expect that as part of a medical visit.” (D04—lower utilizer)

^a^ A higher utilizer was defined as a health professional who, at the time of the interview, had conducted more than 10 video visits. A lower utilizer was defined as a health professional who, at the time of the interview, had conducted 1-10 video visits. The participant ID has a 2-digit number, and either an “A” denoting an advanced practice professional, or a “D” denoting a physician.

Conversely, some respondents found telehealth to augment the patient experience as it provided more frequent follow-up and enhanced convenience, particularly regarding transportation difficulties or when follow-up questions arose after an in-person encounter. Several respondents also viewed telehealth as having the potential to improve the family’s experience of care by providing additional educational sessions to supplement in-person encounters and involving family members in video visit conversations who otherwise would be unable to attend.

### Perceptions of Access to Care

Respondents reported several ways in which they felt telehealth improved access to care (Table 4). One noted that for patients living far from large, comprehensive cancer centers, telehealth allowed them to receive treatment locally while remaining under the care of experts who specialize in their type of cancer. Furthermore, for patients with responsibilities at home, such as caring for children or elderly parents, telehealth increased their ability to see their oncologist. One respondent felt that telehealth allowed for concerns to be addressed in real time and increased availability of in-person appointments for critically ill patients. However, another respondent felt that addressing acute issues via telehealth, rather than an office visit, could lead to delays in important interventions, such as hospital admission.

**Table 4.** Health Professionals’ Perceptions of Access to Care

Theme	Quote (Participant ID—Telehealth utilization status^a^)
Positive	
Patient responsibilities limiting ability to go to in-person visits	“I have a couple of patients who are the primary caregiver for other people in their family, so a parent with dementia, or have small children. And so, for those patients, too, I think getting away from the house is challenging and also really costly, because they have to find other caregivers for those people during that time. So, allowing them to be at home for their visit is … helps them with both of those things.” (D03—higher utilizer)
Access to large cancer centers	“We have patients that come from two hours away, three hours away to get comprehensive care here in a large cancer center. And they get the expertise of people that are highly experienced in their specialty, but they don’t physically have to come here all of the time.” (A06—no telehealth experience)
Allows concerns to be addressed in real-time, may open up health professional schedules to see acutely ill patients in person^b^	“I mean, I guess maybe if scheduling was tight. Not to say that one patient is more important than the other, but say you have this sickly, acute patient that can only come in at this time and you already have a patient scheduled and they’re not willing to come in any earlier or any later. In that case you can maybe offer … would you want to maybe do a video visit here. … But at least any issues or concerns could at least be discussed in real time at that moment and then that obviously sicker patient could then get slotted.” (A08—no telehealth experience)
Negative	
Acute issues may need rapid admission, which can be done more easily if the patient is already in the office^b^	“In stem cell transplant, if you think there’s something acute going on, you need to see them in person, because you need to … you have to decide whether they need to be admitted or not. And if they need to be admitted, you’d rather have them there to be admitted than two hours away.” (D14—higher utilizer)
Language barrier	“I also have a lot of patients that don’t speak English. And that makes it impossible because we need … I need my phone to translate. And it’s hard enough to have a conversation through the translator in the same room, let alone through video. So, it’s just another added layer of difficulty.” (D04—lower utilizer)
Older patient population lacks comfort and skills setting up and troubleshooting technology	“A lot of our patients have trouble using it because I work with a very older patient population. Also, I have a lot of people that are low income and have poor health literacy, so getting the software set up on their phone can be difficult.” (D04—lower utilizer)
Socioeconomic status restricting access to technology	“The people with less SES [socioeconomic status], less financial resources, are gonna be the ones that probably could benefit the most but also have the least likelihood of having access to it. Unless the cancer center was gonna give out some form of a device for them to use at home.” (D08—lower utilizer)
Older patient population lacks devices necessary	“A surprising number of our patients, particularly elderly patients, don’t have access to an appropriate device to do [video visits].” (D19—lower utilizer)

^a^ A higher utilizer was defined as a health professional who, at the time of the interview, had conducted more than 10 video visits. A lower utilizer was defined as a health professional who, at the time of the interview, had conducted 1-10 video visits. The participant ID has a 2-digit number, and either an “A” denoting an advanced practice health professional, or a “D” denoting a physician.

^b^ A subtheme and associated quote that was identified as both a positive and negative feature of telehealth in oncology.

 Finally, respondents felt as though telehealth visits, and specifically the technology required to perform them, were restrictive for older patients, patients who did not speak English, and for those with limited socioeconomic resources.

### Perceptions of Financial Impact or Cost

Respondents had starkly different opinions regarding the financial impact of care (Table 5). Some reported that the copayment for a telehealth visit was unacceptable to patients who did not view video visits as “real” or equal to in-person appointments. Others felt as though the costs eliminated by telehealth visits, such as parking, gas, tolls, and lost work time made them cost-beneficial for patients. Overall, the ambiguity of insurance coverage status for telehealth and the inability to accurately estimate the copayment cost were negative aspects of video visits identified by several respondents.

**Table 5.** Health Professional Perception of Financial Impact or Cost

Theme	Quote (Participant ID—Telehealth utilization status^a^)
Positive	
Eliminates costs^b^	“I think for [Hospital 1] and [City 1 in PA], it could cost them $25 to park. I mean, I love it that they don’t have to park. And then the other financial thing that’s beneficial for these people is if they can … if they have a office or some place they can shut the door, then they don’t lose worktime or use vacation time. So I think it’s great—a video visit. I think that they save money on gas, on parking and possibly on less worktime lost. So that’s where I think it’s beneficial for those patients.” (A05—higher utilizer)
Negative	
Patients feel that they should not have to pay for a video visit^b^	“I think, for the patients that may be the biggest issue is how it’s billed to the insurance company, yeah. And that they may feel that coming here and paying 80, 90, or however many dollars that it may cost, they may feel that it’s not a real visit, but it is, but some people feel that way, I’m not actually in the office seeing the doctor so I should not have to pay.” (A04—lower utilizer)
Ambiguity of insurance coverage status	“[Patients] often ask me what’s my copay or what’s gonna happen here and when I approach our practice manager and others, I get no clear answers because no one seems to have them.” (D16—higher utilizer)
	“What I worry about so much is that it’s never clear to me what the patient is getting charged for the video visit ... it’s not knowing what the upfront cost is for the patient that I absolutely hate.” (D12—higher utilizer)

^a^ A higher utilizer was defined as a health professional who, at the time of the interview, had conducted more than 10 video visits. A lower utilizer was defined as a health professional who, at the time of the interview, had conducted 1-10 video visits. The participant ID has a 2-digit number, and either an “A” denoting an advanced practice health professional, or a “D” denoting a physician.

^b^ A subtheme and associated quote that was identified as both a positive and negative feature of telehealth in oncology.

## Discussion

In this single-center, qualitative study we examined medical oncologists’ perception of the benefits of and barriers to telehealth video visits for patients. We found that respondents often had conflicting opinions regarding the clinical efficacy, quality of patient experience, accessibility, and financial impact of telehealth. Health professionals’ perceptions of telehealth and of the patient experience are critical, as previous research has demonstrated that health professional acceptance of telehealth is fundamental to telehealth adoption.^19^ Concerns regarding the clinical efficacy of a telehealth physical examination are the most commonly reported challenges for the virtual management of cancer during the COVID-19 pandemic.^20^ Our institution offers training in performing a virtual physical examination; however, to our knowledge no research or practice advisories have been published regarding the technique of a virtual physical examination for cancer care. Future research regarding the efficacy of the virtual physical examination, as well as practice recommendations, are necessary given the rapid rise of telehealth for oncologic care.

While recognizing the convenience of telehealth, many respondents emphasized the importance of the health professional-patient relationship in oncology and voiced concerns that patients would feel unsupported, particularly regarding serious or bad news delivery. While our study demonstrated that many respondents felt there was a reduced health professional-patient bond that resulted in a worse patient experience compared with in-person visits, this perception is not universal. Previously, telehealth has been suggested as a means to facilitate a patient-centered care approach that decreases the anxiety associated with an in-person consultation regarding bad news in oncologic care.^21^ This perspective is supported by a study of 351 patients with cancer that found the most important elements for patients receiving information about their cancer were factors related to content, including the physician’s knowledge or competence. Elements related to support—including factors such as being comforted or showing concern; and facilitation, which included being told in a private setting or in person—were rated lower, but still regarded as important by patients.^22^ Notably, this study, among others, recognized the individual preferences of patients and found that these preferences have been associated with certain demographic factors such as gender, age, education, and patient faith in their physician.^21,23^ Patient acceptability of telehealth regarding the delivery of bad news is still largely unknown. However, our study indicated that many health professionals find this mode of delivery to be inappropriate. Future research is necessary to examine how patients view the acceptability of telehealth in regards to receiving bad news or complex information about their oncologic care.

It is important to note that all but 1 interview was conducted between October 30 and November 22, 2019. Therefore, this study is limited in its ability to assess current perceptions of medical oncology health professionals, whose views of telehealth may have changed after a surge in use during the COVID-19 pandemic. Our cancer center’s internal report showed an increase in telehealth appointments, from 1.0% of all visits in February 2020 to 52.4% in April 2020. To assist in the rapid expansion of video visit use necessitated by COVID-19, our institution developed an oncology telehealth task force. The task force facilitated patient enrollment in the video application system, triaged and scheduled appointments, and coordinated technology test visits prior to video visit appointments. Although the formal impact of the task force has not been reported, it is possible that its efforts mitigated previously identified challenges in video visit use. In order to investigate the changing perceptions of telehealth during the COVID-19 pandemic, we have an ongoing study reevaluating oncology health professionals’ insights at our institution.

Many respondents found the copayment associated with telehealth and overall ambiguity regarding coverage of telehealth to be major limitations to their use. According to the Center for Connected Health Policy, as of April 30, 2020, in response to COVID-19, several private insurers, including Aetna, Cigna, and BlueCross BlueShield, announced that they would offer telehealth services at no charge to the patient during the pandemic.^24^ Pennsylvania Medicaid announced that health professionals should bill the same for video visits and in-person visits.^25^ Although Medicaid did not officially change its cost-sharing requirements for its beneficiaries, it did allow health professionals to reduce or waive cost-sharing for telehealth visits without the risk of facing administrative sanctions.^26^ Furthermore, Medicare waived some prior restrictions to accessing telehealth to allow any beneficiary, not only those in rural areas, to receive telehealth services, to do so from their homes, and to use a smart-phone in lieu of other equipment such as computers or tablets.^27^ While it remains unclear how long these changes will last, COVID-19 has ushered in a new era for telehealth services, and further research regarding the barriers to offering them due to perceived financial impact is necessary.

## Limitations

This study had several limitations. All participants were health professionals at a single, urban, academic cancer center, and thus results may have limited transferability to different geographic settings. In addition, the majority of respondents (48.3%) practiced liquid oncology, treating tumors such as leukemia and lymphoma. This may have disproportionately represented perceptions unique to treating these cancer types, which in 2019 accounted for only approximately 10% of all new cancer cases in the US.^28^ However, our study did have a representative sample of health professionals based on gender and racial diversity in oncology, as 15 of 29 participants (52%) were women, and 2 of 29 (7%) were Black. According to the American Society of Clinical Oncology, 32% of oncologists are women and 2.3% are Black.^29^ Although initial recruitment was conducted by 2 authors working at the cancer center (N.H. and A.B.), all subsequent communication with participants and recruitment was conducted by nonclinical team members (A.G., R.G., and A.H.) to minimize participation bias.

Though this study demonstrated health professionals’ perceptions of the barriers to and benefits of telehealth for oncologic care, further research is necessary. Perceptions of telehealth acceptability, including specific benefits and disadvantages, may have changed given the rapid expansion of telehealth secondary to the COVID-19 pandemic and the establishment of the medical oncology telehealth taskforce at our institution. Furthermore, it is critical to directly examine patient perceptions of telehealth and whether the patient experience of telehealth, as well as its potential limitations and benefits, align with those of health professionals.

## Conclusions

This study demonstrated the conflicting opinions of medical oncology health professionals on telehealth and provides insight into potential barriers or limitations to its utilization, as well as the benefits of this health care delivery modality. More specifically, our results emphasize the need to address oncology patients’ access to telehealth technology, especially for older populations, and the acceptability of delivering serious or bad news as telehealth continues to change the landscape of patient-health professional interactions. This is especially relevant during the COVID-19 pandemic, as many institutions worldwide have needed to create or expand telehealth programs.

## Notes

Supplement. **eAppendix.** Interview Guide to Elicit Physician Perceptions of Video Visits
