# Supplementary material for: Medical Oncology Professionals’ Perceptions of Telehealth Video Visits
Source: JAMA Netw Open. 2021 Jan 14;4(1):e2033967. doi: 10.1001/jamanetworkopen.2020.33967 (PMC7809588; doi:10.1001/jamanetworkopen.2020.33967)
Supplement: Supplement. — eAppendix. Interview Guide to Elicit Physician Perceptions of Video Visits [file jamanetwopen-e2033967-s001.pdf]

Supplemental Online Content

Heyer A, Granberg RE, Rising KL, et al. Medical oncology professionals' perceptions of telehealth video visits. *JAMA Netw Open*. 2021;4(1):e2033967. doi:10.1001/jamanetworkopen.2020.33967

eAppendix. Interview Guide to Elicit Physician Perceptions of Video Visits

This supplemental material has been provided by the authors to give readers additional information about their work.

© 2021 Heyer A et al. *JAMA Network Open.*

eMethods: Interview Guide to Elicit Physician Perceptions of Video Visits

1) What do you think about video visits?
    a) When do you think they can be useful?
    b) For what scenarios or patient needs can they serve as an adequate replacement for an in-person visit?
    c) What is convenient about them?
    d) What is inconvenient about them?
2) Access
    a) Have you performed any video visits over the last year?
    b) If not, why not?
    c) If yes, did you need help setting up the visits? Do you find them easy to use?
3) In what scenarios do you offer a patient a video visit?
    a) For which patients (or complaints) do you offer them?
    b) Do patients have trouble setting up the visit?
    c) Do patients complain about any difficulties with the technology?
4) Experience
    a) How do you think patients perceive video visits?
    b) How do video visits affect your daily workflow?
    c) What do you like about video visits?
    d) What don't you like about video visits?
    e) In your experience, how reliable is the technology?
5) Barriers
    a) What do you perceive to be the primary provider barrier(s) for using video visits?
    b) What do you perceive to be the primary patient barrier(s) for using video visits?
6) Effectiveness
    a) What do you think are the relevant benefits of a video visit over an in-person visit?
    b) What are the disadvantages of a video visit over an in-person visit?
7) Cost
    a) What concerns do you have about financial compensation for video visits? RVUs?
    b) What other concerns do you have about cost or compensation related to video visits?
    c) What ways do you think video visits may be cost beneficial to patients?

© 2021 Heyer A et al. *JAMA Network Open.*
